# The Impact of Colleague Suicide and the Current State of Postvention Guidance for Affected Co-Workers: A Critical Integrative Review

**DOI:** 10.3390/ijerph191811565

**Published:** 2022-09-14

**Authors:** Hilary Causer, Johanna Spiers, Nikolaos Efstathiou, Stephanie Aston, Carolyn A. Chew-Graham, Anya Gopfert, Kathryn Grayling, Jill Maben, Maria van Hove, Ruth Riley

**Affiliations:** 1 School of Health Sciences, University of Surrey, Kate Granger Building, 30 Priestly Road, Surrey Research Park, Guildford GU2 7YH, UK; 2 School of Nursing and Midwifery, Institute of Clinical Sciences, College of Medical and Dental Sciences, University of Birmingham, Birmingham B15 2TT, UK; 3 Samaritans, The Upper Mill, Kingston Road, Ewell, Surrey KT17 2AF, UK; 4 School of Medicine, Keele University, Newcastle ST5 5BG, UK; 5 Department of Health and Community Sciences, University of Exeter Medical School, Exeter EX4 4PY, UK; 6 NHS Employers’, 2 Brewery Wharf, Leeds LS10 1JR, UK; 7 University of Exeter Medical School, Exeter EX4 4PY, UK

**Keywords:** suicide, postvention, impact, loss, grief, bereavement, colleague, co-worker, guidance, review

## Abstract

People bereaved by suicide are affected psychologically and physically and may be at greater risk of taking their own lives. Whilst researchers have explored the impact of suicide on family members and friends, the area of colleague suicide has been neglected and postvention guidance for supporting surviving colleagues is often poorly developed. This critical integrative review explored the impact of colleague suicide on surviving co-workers and reviewed postvention guidance for workplaces. Systematic searches found 17 articles that met the inclusion criteria. Articles were appraised for quality and extracted data were analysed using a thematic network method. Article quality was moderate. Two global themes were developed: impact of a colleague suicide comprised themes of ‘suicide loss in the workplace’; ‘professional identities and workplace roles’; ‘perceptions of professional uniqueness’; and ‘professional abandonment and silencing’. Postvention following a colleague suicide comprised ‘individualised responses’; ‘the dual function of stigma’; and ‘complex pressure on managers’. A unifying global network ‘after a colleague suicide’ describes the relationships between all themes. A series of disconnects between existing postvention guidance and the needs of impacted workers are discussed. This review demonstrates the need for robust, systemic postvention for colleagues impacted by the complex issue of colleague suicide.

## 1. Introduction

Suicide is one of the leading causes of death around the world, with more than 700,000 people dying by suicide every year [1]. This means that 1% of all global deaths are due to suicide [2]. Suicide affects the physical and psychological health of the bereaved [3] and, compared to other causes of sudden death, those bereaved by suicide report higher levels of rejection, shame, stigma, and a need to conceal the method of death [4,5]. Every suicide impacts approximately 80 [6] to 135 [7] people, of which 1 in 30 may be deeply impacted and so can be considered bereaved [8]. Suicide bereavement has also been identified as a risk factor for attempted suicide [9,10,11]; approximately 7–9% of people bereaved by suicide subsequently attempt suicide [11,12]. There is also an association with occupational dropout [13].

Previous studies have measured and explored the impact of suicide on family members [14,15,16,17], friends [18], teachers [19], university staff [20,21], therapists, and other healthcare workers [22,23,24]. A recent UK-wide survey on the impact of suicide [4] found that 2% of participants reported being bereaved by a colleague’s death. However, the impact of colleague suicide has not been widely investigated, even though the suicide rate in the UK is higher for certain professions; approximately 12 deaths per 100,000 were suicides between 2011 and 2015 while the risk of suicide for female healthcare professionals was 24% higher than this national average [25].

The support offered to those impacted by suicide is known as postvention [26,27,28,29]. Effective postvention has been shown to improve mental health and grief-related outcomes [30], help bereaved people seek and share support and information, and memorialise their loved ones [31]. While there is some postvention guidance for workers impacted by colleague suicide, see e.g., [28,32,33], existing guidance is limited and is not always evidence based.

Additionally, we argue that suicide research often focuses on the individual rather than the context in which suicide happens [34,35] or on preventing more deaths rather than improving quality of life [36,37]. Critical suicidology, an approach which considers the context and cultures in which suicides happen, such as the occupational factors or antecedents [35,37,38,39], provides a useful lens through which to explore the impact of suicide and support needs of those bereaved. We used this to inform our analysis.

Our review has drawn together empirical research and current guidance on colleague suicide, highlighting what we already know and what the gaps in the research are, signposting the next steps for researchers and support.

### Review Aims

Three questions guided the review:

1. What is the impact on staff of a colleague death by suicide?
2. What is the current guidance for providing postvention support to staff following a colleague suicide and has this guidance been evaluated?
3. What kinds of postvention have been offered, or ought to be offered, to staff affected by a colleague suicide and what are staff experiences of that postvention?

## 2. Methods

An integrative review is a robust methodology [40] that allows a comprehensive understanding of a topic via the synthesis of all available evidence [41]. It is suited to reviewing a combination of diverse methodologies, including experimental and non-experimental research [42], and allows a broad sampling frame [43]. We followed the five steps set out by Whittemore and Knafl [42]: problem identification, through which we developed our review questions; literature search; data evaluation; data analysis; and presentation of conclusions.

### 2.1. Eligibility Criteria

We were interested in reports of the impact of colleague suicide, postvention guidance for workers, and evaluations of that guidance. The eligibility criteria are shown in Table 1.

### 2.2. Search Strategy

The databases listed in Table 2 were searched for the below terms between October and November 2021. The selection process ended in May 2022.

The reference lists of chosen papers were hand-searched for further relevant articles. We did not apply any search limiters in terms of dates or country as we wished to scope the literature as widely as possible.

### 2.3. Article Screening

**Figure 1:**
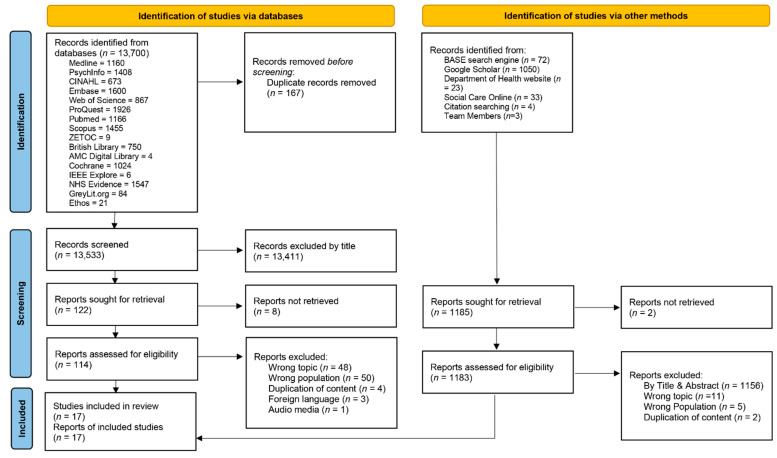
PRISMA flow diagram of the selection process. Adapted from the preferred reporting items for systematic review and meta-analyses (PRISMA) flow diagram [44].

HC and JS independently screened all article titles, rejecting those that did not fit the criteria. We used the Rayyan.ai platform to support article screening. Duplicates were deleted. HC and JS accepted or rejected articles based on their abstracts. Disagreements were discussed until consensus was reached; had we not reached a consensus, a third reviewer (NE) was available to make a final decision. The full articles were read and any which did not fit the inclusion criteria were rejected. Seventeen articles were included in the review. The screening process is summarized in the PRISMA diagram in Figure 1.

### 2.4. Quality Appraisal

Our purpose in appraising the quality of the included articles was not to exclude any articles that could usefully contribute to answering the review questions [45,46] but instead to be aware of the overall quality of the papers.

HC and JS appraised the 17 included articles using a range of tools, including the AGREE-II tool [47], Joanna Briggs Institute (JBI) qualitative appraisal tools [48], the Mixed Methods Appraisal Tool (MMAT) [49], and the Quality of Survey Studies in Psychology (QSSP) tool [50].

HC and JS conducted independent assessments of the quality of all papers, providing inter-rater reliability to check each other’s assessments. Any disagreements were discussed and resolved.

### 2.5. Data Extraction

Data were extracted to meet two aims. Firstly, HC extracted data from all articles to inform an overview of the article attributes. These data are reported in Table 3 and Table 4.

Secondly, HC and JS extracted relevant primary data, author opinion or interpretation, and any other major findings, such as links to existing theory, into a matrix for analysis. Data were organised under the headings impact of colleague suicide on staff members; postvention guidance; and evaluation of postvention guidance. We then uploaded extracted data into NVivo for coding.

### 2.6. Data Analysis

Data were analysed following the thematic network method [64]. Thematic networks are ‘web-like illustrations’ that summarise themes and relationships between themes. We took the following steps as set out by Attride-Stirling [64]:

#### 2.6.1. Coding the Material

HC and JS devised a coding framework based on the research questions and the critical suicidology literature [37,39]. Using NVivo, meaningful sections of the data were coded into that framework [64], which was discussed and refined as analysis continued.

#### 2.6.2. Identifying the Themes

Codes were refined and grouped into similar themes. This resulted in the final table of basic, organising, and global themes (see Table 5).

#### 2.6.3. Constructing the Networks

HC and JS constructed two thematic networks (‘impact of colleague suicide’ and ‘postvention following a colleague suicide’), which can also be considered as a single network under the unifying global theme ‘after a colleague dies by suicide’. Networks were constructed by considering relationships between the three levels of theme.

#### 2.6.4. Describe and Explore the Thematic Networks

HC and JS used the networks as a springboard to fully explore the concepts, connections, and findings arising from the analysis. The upshot of this discussion can be seen in the results section of this paper.

#### 2.6.5. Summarize the Thematic Network

A summary of the thematic networks can be seen in the following section.

#### 2.6.6. Interpret Patterns

Patterns across the two networks were identified and developed during the writing of the results section.

## 3. Results

### 3.1. Quality Appraisal of Included Articles

Despite some high and medium scores, we found that many of the papers had important methodological flaws. Regarding the guidance articles, which were assessed using the AGREE-II tool [47], authors [33,60,61] did not always consult with the target population. Austin and McGuinness [32] drew on case studies but did not report their sources. Only Kinman and Torry [28] and Samaritans [63] reported systematic methods of searching for evidence while no authors reported criteria for selecting evidence or described the strengths and limitations of their evidence. It was not always clear how recommendations were reached [32,33,60,61], recommendations were sometimes not evidenced [60,61], and work was not always peer-reviewed [33,60,61]. Only Berkowitz [33] and Kinman and Torry [28] considered facilitators and barriers to carrying out recommendations. Only Samaritans [63] provided any information about funders.

Regarding the empirical and other articles, six [36,51,54,56,57,58] were appraised using the JBI checklist for qualitative research [48]. All demonstrated congruity between the research methodology and question, although only three [51,56,58] stated the authors’ philosophical perspective. Kleespies et al. [54] were unclear in reporting their methodology and offered little interpretation of their results; Pak [57] presented a ‘conceptual model’ but with no clarity on whether the model was constructed in response to review findings; and Sever and Ozdemir [58] reported their findings in a descriptive rather than interpretative style. Only two papers [51,58] addressed researcher influence on the research. Participants’ voices were well represented by all authors except Gulliver et al. [36], who did not include any verbatim quotes and Kleespies et al. [54], who reflected on rather than reported their findings. Kleespsies et al. [54] were the only authors not to make any ethical statement while Gulliver et al. [36] and Małecka [56] offered no evidence of ethical approval for their research. Conclusions were clearly drawn from the analysis or interpretation of the data in all articles.

Two articles [53,55] were appraised using the JBI checklist for text and opinion [48]. They both met all criteria, although it was unclear whether the stated position of either article was the result of an analytical process. Carr [52] was appraised using the JBI checklist for a case study [48]. All criteria were met at least in part, although it is worth noting that this appraisal checklist assumes that the case study is a medical one, so criteria had to be interpreted broadly to accommodate the nature of the article. Overall, the literature appraised using JBI checklists was of mixed quality, but all met our requirement of making a useful contribution toward answering our review questions.

### 3.2. Thematic Network Analysis

**Figure 2:**
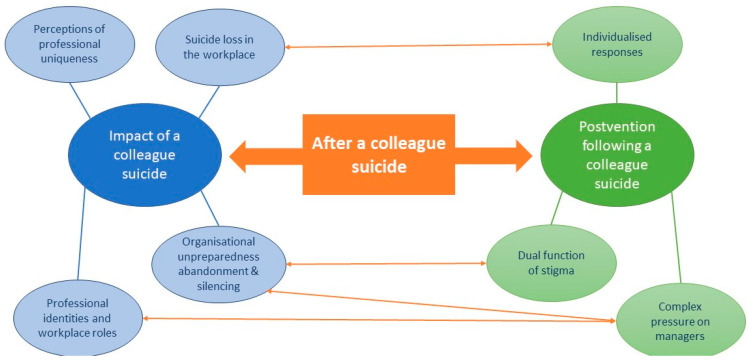
Thematic network.

Our analysis resulted in the development of seven organising themes that sit within two global themes, as illustrated in Table 5. We identified several connections between and across the two global themes, which led to the development of a unifying global theme, ‘After a colleague suicide’. The relationships and connections between the organising, global, and unifying global themes are illustrated in Figure 2.

#### 3.2.1. Global Theme 1: Impact of the Loss of a Colleague to Suicide

##### Suicide Loss in the Workplace

The suicide of a colleague impacts individuals in a variety of ways and brings the usually private process of bereavement into a professional realm. Intense, complex emotions, such as sadness, anger, shame, and guilt [63], may be experienced and heightened by the manner of death:

*Bereavement after suicide is often called ‘grief with the volume turned up’.*

[63]

The most frequently reported emotions following colleague suicide are shock [28,32,52,53,55,56,58,62,63] and anger [28,52,55,57,63]. Anger may lead to further feelings such as ‘confusion, anxiety and shame’ that arise from perceptions of anger as an inappropriate response [28].

Additionally, several authors [28,60,61,62] report behavioural responses, including altered eating and sleeping habits and a need to talk about the event [28]. Some behaviours may be visible and impactful within the workplace, such as absenteeism, presenteeism, or problem drinking [28].

This combination of responses may contribute to the challenging work of grief [32]. Managers must understand that staff are not only experiencing the loss of a colleague by suicide but are also working through the multiple elements of that experience and will require space, support, and empathy. Workers in a 1993 study cited by Lynn [55] (p. 462) expressed the intensity of their experience:

*as the same emotional burden experienced after the death of a family member*.

[65]

Colleagues may feel they must ‘carry on’ after the suicide. This may be a positive way of getting ‘back to normal’ [61] or may present tension between the need to keep working and the need to grieve. For instance, Kinman and Torry (p. 6) note that the performative ‘effort’ of meeting workplace expectations and behaviours ‘can be exhausting and compound grief reactions’ [28]. A colleague’s suicide may give rise to suicidal thoughts or behaviours:

*Sometimes the rationale for this increase in suicide or suicidal behavior occurs out of guilt, a distorted sense of loyalty, or a perceived false “permission” to do so.*

[60] (p. 3)

This is a particularly serious outcome. Leaders and managers ought to be aware of, and alert to, this potential risk. There is a danger that the ‘carry on’ narrative may detract from and indeed hide the real pain and suffering that some staff members may experience.

##### Professional Identities and Workplace Roles

Dilemmas arise when staff come face to face with loss, trauma, and grief whilst inhabiting their professional identities. Specific characteristics of job roles might bring colleagues into contact with dying or recently deceased colleagues, such as deployed military personnel [52] or ambulance staff who may have been called to an incident involving their colleague:

*the ambulance staff that attend the scene could have additional needs in relation to their efforts to help their colleague. There may have been a resuscitation attempt, for example. This places an increased burden on the clinicians present.*

[63]

Professionals may feel they are attempting to navigate dual roles following a suicide. For example, doctors ‘may experience dissonance’ [28] (p. 6) between the roles of ‘healer’ and colleague of the deceased. These dual roles may be especially challenging for team leaders or managers:

*As the line manager, when a colleague dies suddenly you have a responsibility to all team members to assist them in coming to terms with the sudden death, whilst dealing with your own emotions*.

[62]

A sense of impossibility is evident, as leaders report that no matter which approach they take, it is impossible to meet all needs, especially given the challenges of information containment in the social media age:

*It was already on social media, but the senior manager said it wasn’t our place to tell colleagues, as the family may not know yet, so then you’re chastised by staff for not letting people know. […] It all got very messy. And all that was on me. It was a lonely place that day*.

[63] (p. 22)

Further, leaders’ responsibility to safely contain teams is highlighted by Pak [57], who discusses the broad roles that army captains play in nurturing commitment, trust, and good morale within military units.

Questions may be raised here around responsibility, not only for looking after staff following a colleague suicide but also for the colleague who took their life. The suggestion that a colleague suicide may be seen as a failure of leadership [57], potentially triggering mistrust, is a stark reminder that leadership is about creating and nurturing the cultures within which staff work.

##### Perceptions of Professional Uniqueness in Bereavement

Many authors [28,32,52,56,57,58,63] report on the experiences of certain professional populations or participants with specific traits, beliefs, or cultural values. Throughout these reports are perceptions of being ‘unique’ amongst the wider population of those impacted by suicide, making the experience of colleague suicide somehow harder to bear. Pak [57] describes a combination of setting and relationships to explain perceptions of heightened impact:

*military suicide may have an even greater impact than bereavement experienced in most collegial relationships due to the proximity and intimacy required for a unit to function in a combat environment … It is not uncommon for service members to refer to one another as “brother”, “sister”, “brother-in-arms”.*

(p. 189)

Interestingly, Lynn [55] also cites ‘proximity’ and shared experiences as ‘unique’ characteristics of healthcare workers’ roles. Kinman and Torry [28] focus on the nature of small cohesive teams that nurture friendships for GPs. Finally, the shared professional identity and sense of ‘family’ is suggested as the reason for a ‘deeper’ impact on paramedics than others [63].

Perhaps this sense of kinship and shared identities explains professional groups’ notion that their experience of colleague suicide is unique. Colleagues may struggle to articulate who it is they have lost; the deceased is more than a colleague but not a family member. There are challenges here for organisations and leaders in understanding the nature of the loss they are supporting staff to come to terms with. Again, the complexity of loss and need sits uncomfortably within the ‘carrying on’ narrative.

Personal beliefs and cultural norms can also shape a colleague’s ideas about and responses to a death by suicide; diverse belief systems may be held by colleagues who work closely together [55]. Dominant discourses within belief systems may present colleagues with additional challenges:

*“You feel the closeness of death, as in every funeral. However, as a Muslim, I do not find this right. According to our religion, it (suicide) is a rebellion against God.”*

[58]

As Sever and Ozdemir [58] note, it is complex for individuals and leaders to understand and accommodate a range of belief systems. This poses the question of how diverse belief systems might be accommodated within teams who are impacted by a colleague suicide.

##### Professional Unpreparedness, Abandonment, and Silencing

Organisational unpreparedness for responding to a colleague suicide due to skill and knowledge gaps shapes staff members’ experiences, leading to perceptions of unmet needs:

*“It was very surreal–I had to deal with all of this, and I just acted on instinct. There was no help or guidance given to me. Suddenly I was in charge of everybody else’s feelings and just expected to carry on as normal.”*

[28] (p. 7)

Unpreparedness may take the form of skill and knowledge gaps or lack of guidance:

*In the absence of any guidance, our interviewees were obliged to ‘ring round’ desperately hoping to receive help which was not forthcoming. This clearly intensified their distress and the difficulties that practices, especially small practices, experienced*.

[28] (p. 17)

Placing the onus on individuals to seek support to meet their individual needs assumes that people experiencing shock, anger, guilt, and grief can identify and articulate what those needs may be. Whether these staff members knew what they needed, they knew they needed something, and the lack of resources within their organisation led to a wider search.

Małecka [56] reports that similar deficits were experienced in a Higher Education setting, where a colleague’s suicide went unacknowledged, leaving staff members feeling abandoned, confused, and angry. Kinman and Torry [28] also report a ‘reluctance’ toward responding to need. It is unclear what drives this reluctance; perhaps not knowing how to respond or fear of doing it ‘wrong’.

Yentis et al. [59] demonstrate the stark difference between the numbers of bereaved anaesthetists who felt supported (*n* = 22) and those who did not (*n* = 179):

*[participants] described absent or poor support and in some cases, deliberate attempts to prevent or stifle discussion and/or debriefing, although in some cases the issue of protecting the deceased’s confidentiality and/or sparing the family further anguish was mentioned.*

[59]

There is evidence here of a process of silencing, where no platform for acknowledging or discussing needs is provided. Bogle [51] reports the perception of US law enforcement officers who describe the administrative response to their colleague’s suicide as ‘avoidant’:

*As long as you’re doing your job, doing what you need to do and say, administration would acknowledge if you lost your life in the line of duty. You’d be a hero. But the moment [an officer] loses their life because of suicide, it’s unspoken.*

[51] (p. 97)

A similar response is reported by Belgian military service members [53], whose perceptions of social stigma act to silence them, thus further perpetuating the cycle of stigma. Silencing stigma is also noted by Małecka [56] in a Polish Catholic university; note that in Catholicism, suicide is considered a mortal sin. The conspiracy of silence experienced by these police officers, service members, and academics across organisational, social, and cultural contexts denies them opportunities to honour their deceased colleagues and process the impact of the suicide and risks psychological wellbeing [53]. Ultimately, silencing and stigma leave impacted staff alone with their need to find meaning and answers following their colleague’s suicide.

#### 3.2.2. Global Theme 2: Postvention Following a Colleague Suicide

##### Individualised Responses

Currently, postvention guidance tends to focus on individuals and individual change rather than contexts and systemic change. Attempts to consider the context within which suicidal behaviours occur are often lacking. We see this as a flaw in existing guidance.

A common claim was that personal vulnerabilities and mental health challenges increased the risk of contagion, whereby one death by suicide increases the risk of subsequent deaths by suicide among those who are affected [28,53,56,61,63], with no consideration of context. The following quote from a police officer [51] demonstrates a deep-seated belief that suicidal feelings are solely located within the individual:

*‘We’re all adults. You have entrusted us with the authority to take people’s freedom and the authority to take lives, if necessary. […] So aside from offering programs, there’s nothing anybody can do to stop them’.*

[51] (p. 106)

While this participant describes the culture in which the suicidal behaviour is occurring, they still feel the only available option, on which they place little worth, is individual support.

Strategies for communication about the reasons for suicide were also individualised:

*the important information is that the person mistakenly felt that they could not get help for his or her problems, when in fact help was possible.*

[33] (p. 163)

Since suicide happens within a context, changes to culture (in addition to individual support) may also be beneficial for postvention.

Checklists of postvention tasks [28,32,33,53,61,62,63] or the utilisation of psychological or organisational models of support [28,32,33,53,57,61] similarly tended to focus on individual needs rather than culture.

Training was mentioned as a potential tool for effective postvention. Most proposed education focused on individual needs or signs of mental ill-health [32,33,51,63]. In contrast, Pak et al. [57] suggested training as a way for leaders to positively influence work culture:

*Military leaders can be encouraged and taught to recognize that to compartmentalize the unit suicide and to ignore it in the short-term, may also place their units at risk.*

We endorse positive cultural changes as part of postvention [37] whilst cautioning against putting unrealistic pressure on managers, who may also be grieving or operating within an under-resourced system.

##### Dual Function of Stigma

Stigma both leads to inadequate postvention and arises from it. It leads to inadequate postvention since, if an organisation cannot talk about suicide, it cannot properly support those impacted by it. It arises following poor postvention because, if postvention is steeped in stigma, it perpetuates stigma at individual and organisational levels.

Authors reported that suicide was not properly acknowledged in the workplace [33,53,56,60,61,62]. However, it was widely agreed that this increased risk [28,32,33,57,60,63]. Discussion of suicide helps address stigma and so could aid postvention. However, one could question whether acknowledgement of suicide is enough without also acknowledging any difficulties with the context in which the suicide occurred.

Workers of various professions reported a culture of invulnerability [52,53,56], where mental ill-health was unacceptable. This could prevent education around suicide, impacting postvention:

*‘To have training on officer suicide would mean that [suicide] would have to be talked about. And that’s not going to happen.’*

[51] (p. 96)

This culture may further challenge postvention by preventing workers from being open about emotions or asking for help [51,53].

Organisations operating from a culture of invulnerability could also perpetuate stigma:

*Personnel allowed to attend the service were limited […]. Restricting access to the memorial service created a sense of shame about the death.*

[52]

Additionally, it could be suggested that the term contagion, which was commonly used in reference to the statistic that one suicide may result in more [61,62,63], may perpetuate stigma and so hamper discussion and healthy postvention. Perhaps non-pejorative language such as ‘further suicides’ may be more useful. Given the prevalence of this narrative, it is perhaps unsurprising that contagion continues, and has:

*…sometimes led to misguided efforts to maintain secrecy after a suicide death, including blaming or stigmatizing the deceased.*

[33] (p. 168)

More helpfully, several authors made suggestions for how stigma could be combatted. These include the use of more sensitive terminology [63] and group counselling [58]. Several reported workplace cultures that were already supportive [53,58,63].

##### Complex Pressure on Managers

Complex pressure is placed on managers of workplaces in which a colleague dies by suicide, as the delivery of postvention support becomes their responsibility. Specific tasks that managers might be expected to undertake included regularly checking in with staff, looking out for affected colleagues [28], and being visible to workers [61]. Additionally, leaders may be expected to undergo training to deliver postvention [33,51,61,63].

Managers must also provide practical support such as accompanying employees during inquests [32], offering meals and transport [33], or arranging alternative duties for staff [63]. Some authors provided detailed explanations of ways for employers to emotionally support grieving workers [28,32,53,62,63], such as engaging in empathic listening and sharing stories. Several agreed that leaders must guide employees through the grieving process [55,61,63]. Further pressure on leaders is added by the suggestion that they should be “a role model for healthy grieving” [32,61].

Various authors acknowledged that managers, who are also grieving, must also be supported [28]. Suggestions included covering time off [28,63], regular check-ins with HR [63], and reassurance that it is OK to express emotion [63]. We feel that these are worthy suggestions that may help combat that toxic culture of invulnerability. Working with teams within [52,61] and outside of [28,32,33,53,62,63] the organisation to deliver postvention may also relieve pressure. Such support for managers, who are uniquely pressured following the suicide of an employee, is sorely needed.

#### 3.2.3. Unifying Global Theme

Thus far, we have described two thematic networks: Impact of a colleague suicide and Postvention following a colleague suicide. Both networks are illustrated in Figure 2 in blue and green, respectively. In line with the thematic network methodology [64] (p. 393), this figure is intended to explore and illustrate the deep meaning and relationships behind the reviewed texts rather than to demonstrate causal relationships. We found that both networks describe events that occur simultaneously following a colleague suicide, and that these networks feed into and inform each other. Thus, they can be illustrated as being connected by a unifying global theme: After a colleague suicide, represented in Figure 2 in orange. The orange arrows indicate how individual experiences and needs following a colleague suicide are shaped by the availability and content of postvention support. Likewise, the design and delivery of postvention impact how workers respond to and heal from the suicide of a colleague as illustrated in Figure 2.

Specifically, the impact of suicide loss in the workplace can be heightened or ameliorated by the response of the organisation. Our findings evidence that staff experiences occur within the contexts and cultures of workplace settings, identities, and roles. Guidance, however, promotes individualised approaches to responses that fail to acknowledge these factors. A holistic approach, looking at the context and systems within which the suicide occurred and support for teams and whole organisations, in addition to any necessary individual responses, may reduce overall distress. Further, stigma is linked to organisational unpreparedness, abandonment, and silencing. Teams who are delivering postvention that is marred by stigma will, as our findings demonstrate, find it harder to acknowledge and respond to suicide, leading to a silencing, which, in turn, perpetuates stigma. Solving organisational unpreparedness may fall to managers, adding to their complex pressure; equally, if managers cannot fulfil the unrealistic battery of tasks assigned to them following an employee suicide, the organisation may continue to be unprepared and silence grieving workers. Finally, the organising themes of complex pressure on managers and professional identities and workplace roles are intertwined, as the dual roles that managers must inhabit whilst simultaneously grieving and caring for bereaved employees further add to their complex pressure.

## 4. Discussion

We reviewed and synthesised 17 articles, including empirical studies (*n* = 7), case studies (*n* = 1), opinion pieces (*n* = 2), and guidance (*n* = 7). We explored the impact of a colleague death by suicide across a range of workplace settings; reviewed the current guidance for workplace postvention support following a colleague suicide; and developed an understanding of what kinds of postvention support have been offered, or authors think should be offered to staff affected by a colleague suicide. We found that the workplace impact of colleague suicide and associated postvention has been sparsely explored, and published articles are of an overall moderate quality. Published guidance is rarely underpinned by empirical evidence while the guidance included in this review cite each other (with and without full acknowledgement and referencing). Some guidance appears comprehensive, but it is not always clear where the underpinning knowledge has come from. Experiences of loss and bereavement by suicide were shaped by workplace contexts, cultures, and job-role identities. Further, organisational responses, or lack thereof, created additional struggles for staff.

In this discussion, we explore three (dis)connections between staff experiences of impact following a colleague suicide and the postvention guidance currently available to managers and organisations, as illustrated in our thematic network (Figure 2).

### 4.1. Workplace Cultures, Professional Contexts, and Individualised Responses

Suicide loss is shaped by perceptions of professional identity and workplace settings. However, this is unacknowledged in postvention guidance, which takes an individualised view of cause and impact. Whilst the emotional impact reported by staff following a colleague suicide reflects the wider literature [66,67,68,69], experiencing this impact within professional identities and workplace settings complicates individual responses.

For instance, perceptions of professional identity and uniqueness shape staff experiences of grief. We reviewed the experiences of police officers, firefighters, military personnel, and primary care health professionals. Such professionals may be working within a culture of invulnerability [70], whereby perceptions of being impervious to work-related stresses become part of a professional identity. Staff who perceive themselves as invulnerable are less likely to find psychological safety following a colleague suicide. Researchers have concluded that talking about vulnerability and illness reduces perceptions of isolation and promotes coping mechanisms for GPs [71]. It is likely that such openness may also promote healthy coping in other professions. Furthermore, we found that staff across a range of professions believed that their experience of suicide loss was more impactful due to their perceptions of the unique traits of their job role or professional identity. While several professions perceived themselves as unique for similar reasons, this indicates that these ‘unique’ attributes and their impact on professionals’ experience of a colleague suicide ought to be understood, acknowledged, and incorporated into support to meet staff needs for all groups.

Currently, workplaces do not provide the time and support required by employees to undertake the emotional work that arises following a colleague suicide. We found that a ‘carry on’ narrative dominates, prioritising work tasks and productivity over emotional needs. Similarly, when exploring the experiences of bereaved staff on their return to work, Bento [72] used the phrase ‘the show must go on’ to describe employees’ perceptions of silence or pressure to catch up with work tasks. It may be that leaders working in ‘24/7′ professions such as medicine or the military are expected to keep working to prevent the fallout from a depleted workforce, meaning they must put work ahead of their wellbeing.

Hochschild [73,74] utilises the concept of ‘feeling rules’ to describe the processes of emotion management that occur in workplace settings. Similarly, Doka [75] talks about ‘grieving rules’ that describe societal norms around loss and grief behaviours. Together, these concepts may provide a framework for better understanding how staff are expected to manage grief in the workplace and how organisations operate to direct grieving processes away from the workplace. Within the social model of individualisation, we are expected to do our emotional work in the privacy of our homes [76]. However, when grief occurs at work, this expectation generates further stress and an understandable disconnect for staff. We found that staff must work to navigate these complex expectations surrounding grief after a colleague’s suicide. Similarly, Grandey [77] identified that employees suppress or regulate emotions following a stressful event to deliver an appropriate emotional presentation for the workplace. Such emotional management has been conceptualised as emotional labour [73]. When expressed emotions differ from those that are felt, emotional dissonance and internal tension may result [78]. As such, emotional labour is stressful and may lead to burnout [79].

As Pitimson [80] points out, UK legislation regarding compassionate leave does not recognise the death of a colleague, meaning any leave is at the discretion of the employer. In response to this point and the findings of our review, we argue that time must be offered in workplaces to accommodate the emotional work that may follow a colleague suicide and avoid the risk of emotional burnout.

It would be beneficial for authors of postvention guidance to offer strategies for addressing these specific staff experiences and needs following a colleague suicide. Overall, the guidance we reviewed did not take an organisational perspective, nor did it address professional identity and working spaces as the contexts within which loss and grief must be navigated and postvention support delivered. As previously noted, current guidance has drawn only sparsely on empirical evidence, which may explain this disconnect between need and delivery. We argue that postvention guidance must draw on the lived experiences of the people it aims to support. The reviewed guidance largely misses an opportunity to support organisations and staff by meeting them at the point of their experience.

### 4.2. Unpreparedness, Abandonment, Silencing, and the Perpetuation of Stigma

We found that organisational unpreparedness for suicide loss generates feelings of abandonment and perceptions of silencing that further complicate experiences of grief and perpetuate perceptions of stigma. Managerial or organisational failure to acknowledge colleague suicide and its impact leaves staff feeling abandoned in navigating their path to recovery. Pitimson [80] reports that a lack of workplace acknowledgement leaves bereaved staff with fears of being judged and a need to find safe places at work for privately expressing grief. As Lattanzi-Licht [81] states, the workplace requires the bereaved to be silent, hiding their feelings. Disenfranchised grief [82] refers to instances of dismissal when either the relationship with the deceased, the nature of the loss, or the griever themselves are not recognised. Doka [82] proposes that acknowledgement of grief is necessary for bereavement to be completed. The silence and silencing found in our review leave staff unable to talk about or process their experiences. Thus, the idea of suicide as a taboo [83] topic is perpetuated, staff are unsupported in their grief, and their trauma remains unacknowledged and unaddressed. Our findings illustrate that, alongside the absence of organisational response, the event of a colleague suicide and attempts by staff to mourn and remember their colleague were actively silenced. Staff grief in the workplace is not just disenfranchised; it is actively stifled [84].

This active silencing has an impact. As our findings demonstrated, stigma both leads to and arises following inadequate postvention. When organisations do not deliver postvention, they perpetuate stigma around suicide by failing to provide forums for conversation, acknowledgement, understanding, and healing. Paradoxically, it is the stigma surrounding suicide, and associated fears, that may contribute to organisations failing in this way. There is a fear of acknowledging suicide due to misunderstandings around risk of ‘contagion’ and the likelihood of further deaths by suicide [11]. We argue that it is the role of guidance to address these naïvetés. However, if guidance is not underpinned by evidence, it may be difficult to convey these messages robustly.

### 4.3. Managers: Identities, Roles, and Complex Pressures

Colleague suicide generates complex challenges for managers. This is exacerbated by perceptions of professional identity, whereby managers are perceived as strong and knowledgeable, and further, by postvention guidance, which situates managers as supporters of other staff. Balancing the needs of their team alongside managers’ own needs, expectations that the workplace should continue to function as usual alongside staff grief, and the need to communicate clearly to staff whilst balancing the preferences of the deceased’s family and the need to protect the deceased’s privacy are all factors that generated this complexity. The wider literature also identifies the manager as a key provider of support and comfort when a staff member is bereaved [85,86,87].

Several authors highlight that, with proactive support and compassion, the workplace can facilitate healing following bereavement [85] and that bereaved staff may feel safe in the familiarity of the workplace [80]. Compassion is defined as ‘an active orientation towards the well-being of others who are in pain’ [88] (p. 168). Kanov [89] suggests that managers are well placed to offer compassion by noticing the suffering of others, feeling empathic concern, and acting to alleviate suffering. The manager must be alert, empathically in tune with others, and knowledgeable about appropriate proactive responses. It is often assumed that managers will provide this support and compassion whilst managers’ struggles are not acknowledged [80,85,89].

The agency of bereaved staff within the supportive relationship is recognised by Dutton et al. [90], who posit that compassion requires both parties to interpret and understand each other’s circumstances to make sense of the situation. Even here, however, there is no acknowledgement that the manager may be experiencing their own grief. We propose that, importantly, postvention guidelines provide guidance for the support of managers whilst they, in turn, support their teams. Additionally, guidance can identify external sources of postvention support (the availability of which, we acknowledge, varies), so that the weight of being the expert and supporting staff can be lifted from the potentially grieving manager.

Considering the broader contexts that underpin the complexity faced by managers allows for insights into the competing pressures of meeting staffs’ emotional needs alongside the demand for the business to function. Pitimson [80] notes that individualism and capitalism can shape the experiences of grief in the workplace. Peticca-Harris [86] highlights this in her first-person account of restaurant managers’ responses to the sudden death of a staff member. She describes how the need to keep the restaurant open blinded managers’ ability to see, or relate to, the distress staff were experiencing [86]. Granek [91] suggests that control of grief in workplaces is political in terms of the expectation that staff will continue to contribute within capitalist societies. Peticca-Harris [86] (p. 608) concludes that ‘managers did not know what to do and how to do it, and that brought about shame and embarrassment because it was at odds with the archetypes of leadership that dictate that leaders should just know [92]’.

These multiple juggling acts are addressed in part in postvention guidance, which often suggests the formation of a postvention committee or group, meaning postvention tasks are planned for and shared (see e.g., [62,63]). This may be feasible in a larger organisation. However, it is likely that one team manager would still need to provide information, identify staff who need support, and facilitate the implementation of support resources. In smaller organisations, or those that have not implemented a postvention team, individual managers are likely to have to implement postvention support to team members whilst also having to deal with their own responses to the loss. Effective, evidence-based training may help lessen managers’ load. Attendees of postvention training for clinicians who support parents following the suicide of a child reported increased knowledge, skills, and confidence following the session [9]. We propose that similar outcomes might be achieved if training was provided to managers and leaders following colleague suicide. However, it must be considered whether such training is available before recommending it as a solution. As Tehan and Thompson [87] acknowledge, managers need to feel knowledgeable, skilled, and equipped.

This is the first review to specifically explore the impact of colleague suicide and related postvention guidance. The robust methodology utilised in this review allowed us to bring together a wide range of source literature and first-person experiences alongside associated guidance. This has enabled us to identify areas of disconnect between experience and response, and make recommendations for improving the guidance and, therefore, the care of staff bereaved or affected by a colleague suicide. A limitation of this review is that we were unable to include papers written in languages other than English, due to time and budget constraints. Additionally, although a comprehensive search strategy was used, we may have missed literature not storied in the searched databases.

We recommend the following steps for practice, policy, and research:

- Physical and emotional time and space for processing and grieving ought to be provided in workplaces to accommodate the emotional work that may follow a colleague suicide.
- Postvention guidance must be developed for specific professional groups, drawing on the lived experiences of that group, so that the specific needs of professional cultures and traits are understood.
- Guidance ought to include education around the dangers of stigma, the misunderstanding or misuse of ‘contagion’, and the protective factors of acknowledging and talking openly about suicide.
- Postvention guidance should acknowledge the competing pressures that managers experience following a colleague suicide and provide guidance for the support of managers whilst they, in turn, support their teams.
- Guidance could offer alternative models to the ‘postvention team’ to accommodate the limited resources of smaller or less resource-rich organisations. This may include accessing external expertise and support, if such a service is available.
- Postvention team members, managers, and team leaders should be offered training around topics such as suicide stigma, risk, and ‘contagion’. This should include strategies for supporting teams and individuals.
- Future researchers may wish to explore the impact of colleague suicide on those with pre-existing mental health conditions (such as military personnel with PTSD).
- Development and evaluation of postvention guidance that is informed by empirical evidence for specific professional groups is needed

## 5. Conclusions

We suggest that colleague suicide can impact workers in healthcare and other settings and that perceptions of grief are complicated by professional identities and workplace cultures. A burden is placed on managers to be knowledgeable, skilled, and available to support staff. Current postvention guidance, and the postvention offered to colleagues, whilst well-meaning, is not evidence-based, takes an overly individualistic view and may perpetuate stigma, and has not often been evaluated. As such, we call for more evidence-based, systemic postvention guidance for workers and managers.

## Figures and Tables

**Table 1:** Inclusion and exclusion criteria.

Inclusion Criteria	Exclusion Criteria
Reported on the experiences of people following a colleague suicide	Reported experiences following the suicide of a client, patient, service-user, student, family member, or anyone outside of work
Reported responses to an incident of, or set out guidance or policy in response to, the suicide of an employee or colleague	Reported only on prevention or intervention of colleague or employee suicide or on causes of suicidal ideation or behaviour
Qualitative, quantitative, or mixed methods research studies, reviewed original data, reported a first-person account, case study, or opinion piece	
Reported or evaluated the implementation of postvention guidance or support programmes for staff	
Published in the English language	
Contributed usefully to addressing the review problem	

**Table 2:** Databases and search terms.

Databases	Grey Literature Databases	Search Terms
Medline	BASE	suicide
PsycINFO	Google Scholar	AND
CINAHL	British Library	[colleague* OR co-worker* OR staff OR personnel OR employee OR workplace]
Embase	Ethos	AND
Web of Science	ZETOC	[postvention OR guidance OR guidelines OR support* OR therap* OR response OR policy OR evaluat*]
ProQuest	AMC Digital Library	
PubMed	Cochrane Library	
Scopus	IEEE Xplore	
	NHS Evidence	
	Social Care Online	
	Social Science Research Network	
	United Kingdom Department of Health	
	GreyLit.org	

**Table 3:** Attributes of the included empirical studies, survey studies, case studies, and opinion pieces.

Author & Date	Location	Type of Study	Study Aim/Research Question	Setting and Participant Details	Data Collection	Data Analysis	Results/Findings
Bogle, C.L. (2018) [51]	US	Qualitative study	Exploring the lived experiences of law enforcement officers concerning colleague suicide and the impact a suicide has on a law enforcement agency/department.	Police Department Law enforcement officers *n* = 11 Male = 7 African American = 10 Caucasian = 1	Semi-structured interviews	Thematic analysis	Four major themes: (1) Uniqueness of the law enforcement community (2) Lack of available resources regarding mental health services (3) Reactive response to the suicide event and (4) The necessity for consistent mental health services.
Carr, R.B. (2011) [52]	Iraq	First-person account	Reporting the author’s first-hand experience of supporting a US army unit in Iraq after a soldier suicide.	US Army Psychiatrist in support role. *n* = 1 Male	N/A	N/A	Acute effects of suicide and effects over the subsequent four months.
Deheeger, J. (2008) [53]	Belgium	Report	Reporting the incidence, impact and postvention response following a colleague suicide in the Belgian Defence.	Belgian Armed Forces No Participants	N/A	N/A	Grief, guilt, and feelings of blame Fear of social stigma The service member’s need for help Postvention trajectory of care: Pre-incident education Post-incident procedure of psychosocial care for victims Structure of the postvention crisis intervention procedure.
Gulliver et al. (2016) [36]	US	Evaluation	To subject the New York City Fire Department (FDNY) standard operating procedure (SOP) to an iterative process to develop a national guideline for suicide postvention.	US Fire Department Initial Expert Review Group: *n* = 5 Female = 1 All = at least two years exp in fire service	Expert review group and 90-min focus groups, video recorded.	Data were taken at face value and used to inform the development of the guidance.	The expert review group discussed the need for more depth in the FDNY SOP as well as adding more information and procedures around responding to family and department members. Focus groups suggested making the SOP more operational and directive and breaking it up into two documents: (1) educational material (to be shortened into a pamphlet) and (2) the SOP. They also suggested calling it a guideline rather than an SOP. Feedback was incorporated into the final version of the SOP guidelines.
				Six focus groups: *n* = 61 75% male Mean age = 47 years 22.9% Hispanic 9.8% African American 72/1% Caucasian	Six focus groups in three test cities reviewed the SOP manual and provided feedback on barriers to implementation		
Kleespies et al. (2011) [54]	US	Literature review, interviews, and review of case reports	To investigate the incidence of psychologist suicide and its impact on colleagues, students or interns, patients or clients and the profession.	US Psychologists. Reports that 14 cases of suicide were identified but does not clarify the number of participants interviewed nor participant details.	Interviews	Not reported	Postvention efforts to address the needs of all survivors are needed. Professionals can help colleagues by clarifying the wishes of the deceased for closing their practice. The extent to which colleagues may experience a complicated bereavement and need support is undetermined.
Lynn, C.W. (2008) [55]	US	Case study	To explore the role of the occupational health nurse in supporting staff following the death co-worker suicide.	US Hospital Health workers	Case Study	N/A	Occupational health nurses can be the guiding force for first-line nurses after a suicide. Preparation begins with recognising that suicide is a genuine health emergency and requires the same planning as any other safety-related issue. Education and resources through EAP can prevent suicide and facilitate postvention.
Malecka, K.A. (2020) [56]	Poland	Qualitative multiple case study	Exploring how four Polish universities managed suicides. Presenting the lived experiences of participants holistically.	Higher Education Institutions Colleagues of deceased HE workers. *n* = 7 Academic staff = 5 Administrative staff = 2 Polish = 5 ‘Of foreign descent’ = 2	Semi-structured interviews	Thematic data explication	Eight thematic areas: Notification of the deaths Personal responses to the deaths Institutional & managerial responses Professional help Formal and informal acknowledgements Administrative matters The reality of organisationally sponsored loss of education Mortality (mis)management: additional loss stories
Pak et al. (2019) [57]	US	Literature review	Providing a summary of the postvention literature with special emphasis placed on the military organisation; proposing a conceptual model for understanding Military-Unit Suicide Survivorship; and highlighting postvention strategies within the DoD.	US Army No participants	Qualitative, narrative, and evaluative review. Methods of data collection and analysis are not reported.		Proposes a conceptual model for military unit suicide survivorship based on the literature. Proposes postvention strategies and recommendations. Makes research, clinical, and policy recommendations.
Sever & Ozdemir (2019) [58]	Turkey	Qualitative study	Exploring the impact of a staff member’s suicide on the organisation, faculty, and administration in a Turkish academic institution. Highlighting the influence of culture and belief in responses to suicide, where many people are Sunni Muslims, a belief system that strongly disapproves of suicide.	Higher Education Institution *n* = 7 Male = 4 Colleagues *n* = 5 Administrators *n* = 2	Open questions provided by email	Interpretative phenomenological design	Two categories, seven themes Personal: Shock Questioning and feeling responsible Stigmatisation or asking for forgiveness Personal lessons Regrets Organisational: Solidarity and administrative support What needs to be changed
Yentis, Shinde, Plunkett & Mortimore (2019) [59]	UK	Survey	A working party to review anaesthetist suicide and provide guidance for anaesthetists, departments, and employers.	Survey sent to anaesthetists working in the UK. *n* = 3638	Anonymous online survey	Descriptive statistics	Most respondents were unaware of the existence of policies on mental illness, addiction, or suicide. 1916 cases of suicide were reported by 1397 respondents. A third of respondents who reported a suicide had experienced more than one case. Most reported suicide in the last 10 years involving anaesthetic drugs. Deficiencies were noted in support and how deaths were handled, although examples of good support were also described.

**Table 4:** Attributes of included guidance.

Authors, Date & Title	Location	Type of Article	Setting	Aims	Evidence Base	Guidance/ Recommendations
Vanderpol & Beyer (2019) [60]	US	Guidance	Construction industry	To share perspectives, strategies, resources, and tools to help contractors respond appropriately to a colleague suicide.	Draws on existing knowledge and guidance. No reference list or evidence base cited.	Defines postvention. Presents key points for critical incident management and strategies to support colleagues following a suicide. Q&A with the authors. Signposts to support websites and articles.
Leading a company in the aftermath of a suicide loss						
Berkowitz et al. (2014) [33]	US	Guidance (book chapter)	Organisations	Not stated	Draws on existing literature.	Concludes: Organisational postvention is recommended, but guidance is sparse. Various factors complicate the work. Postvention should be an evolving process that attends to the guidelines and principles in this chapter. Longitudinal and comparative studies are needed. Qualitative studies are needed to understand need and inform postvention interventions.
Organizational postvention after suicide death						
Austin & McGuinness (2012) [32] Console & The Irish Hospice Foundation	Ireland	Guidance	The workplace	To help organisations increase their understanding and confidence in responding to workplace suicide.	Presents case studies but does not cite sources, so they could be fictionalised. Cites three references.	Provides guidance for employee suicide on-site and off-site, when an employee is affected by the suicide of someone close and when a former employee dies by suicide. Guidance also provided for developing a bereavement policy for dealing with suicide. Signposts to suicide support organisations.
Breaking the silence in the workplace: A guide for employers on responding to suicide in the workplace						
The workplace postvention taskforce of the American Association of Suicidology & the workplace taskforce of the national alliance for suicide prevention. In partnership with the Carson J Spenser Foundation & Crisis Care Network. (2013) [61]	US	Guidance	The workplace	Not stated	Cites a source for their definition of postvention. Cites the Individual Differences Models (Mancini & Bonanna, 2009), the ACT Model (VandePol, 2003) and the CDC definitions of ‘suicide’ ‘suicide attempt’ and ‘suicidal ideation’. However, no evidence base cited to underpin the guidance.	Defines postvention. Presents a three-phase (acute, recovery, reconstructing) approach to delivery. Provides sample comms memos; signposts to resources; provides a decision-making flow-chart.
A manager’s guide to suicide postvention in the workplace: Ten action steps for dealing with the aftermath of a suicide.						
Kinman & Torry (2020) [28] Supporting Occupational Health and Wellbeing Professionals & The Louise Tebboth Foundation.	UK	Guidance	Primary healthcare	Guidelines intended to inform a flexible crisis management strategy that provides information and support to primary care practices at different stages following a colleague suicide. May also be useful to similar small organisations.	In-depth interviews with GPs who have experienced a co-worker suicide, as well as contributions from other stakeholders. Interviews analysed by two researchers independently. A grounded theory approach was used where themes were identified and expanded until saturation. Full list of references included.	Presents postvention guidance for the first day, first week, first month, and longer term. Guidance is presented alongside participant quotes. Presents an Actions Needed summary table. Signposts to other resources.
Responding to the death by suicide of a colleague in primary healthcare: A postvention framework						
Business in the Community; The Prince’s Responsible Business Network; Public Health England; Samaritans (2017). [62]	UK	Guidance	The workplace	Toolkit to help organisations consider the issues that arise from workplace suicide; mitigate the impact of suicide; design a relevant postvention protocol.	Includes case studies from named organisations but does not cite these as underpinning evidence. No evidence base or references included.	Presents chronological guidance: Be prepared When suicide happens Grieving, post-traumatic phase Legacy phase Reflection time Includes signposts to resources and case studies.
Crisis management in the event of a suicide: A postvention toolkit for employers.						
Samaritans & Association of Ambulance Chief Executives (2021). [63]	UK	Guidance	Ambulance service	To help ambulance services, particularly leaders in HR and frontline managers, manage the impact of an employee suicide or attempted suicide on colleagues.	Cites six references that explore: mental health problems among UK ambulance workers; paramedic perceptions of distress, stigma, and utilisation of support services; mental health in the ambulance service; effects of exposure to self-harm on social media study; effects of educative suicide prevention websites; contagion.	Presents chronological guidance: Be prepared Communicating after a suicide When suicide happens Grieving, post-traumatic phase Legacy phase Reflection time Further information and resources.
Ambulance service employee suicide: A postvention toolkit to help manage the impact and provide support.						

**Table 5:** Relationships and connections between the organising, global, and unifying global themes.

Organising Themes	Global Themes	Unifying Global Theme
Suicide loss in the workplace	Impact of the loss of a colleague to suicide	After a colleague suicide
Professional identities and workplace roles		
Perceptions of professional uniqueness in bereavement		
Professional unpreparedness, abandonment, and silencing		
Individualised responses	Postvention following a colleague suicide	
The dual function of stigma		
Complex pressure on managers		

## Data Availability

No new data were created or analyzed in this study. Data sharing is not applicable to this article.
